# Concurrent hepatic cirrhosis and chronic lymphocytic leukemia: a challenging coexistence: a case report

**DOI:** 10.1186/s13256-026-05946-8

**Published:** 2026-04-18

**Authors:** Fatemeh Khodadadpour Mahani, Nazanin Zeinali Nezhad, Reza Ghaderi, Elham Jafari, Mana Khazaeli, Samaneh Omidi Kermanshahaninejad, Behrang Shamsinejad

**Affiliations:** 1 Research Center of Tropical and Infectious Diseases, Kerman University of Medical Sciences, Kerman, Iran; 2 Physiology Research Center, Institute of Neuropharmacology, Kerman University of Medical Sciences, Kerman, Iran; 3 Clinical Research Development Unit, Afzalipour Hospital, Kerman University of Medical Sciences, Kerman, Iran; 4 Pathology and Stem Cell Research Center, Department of Pathology, School of Medicine, Kerman University of Medical Sciences, Kerman, Iran; 5 Gastroenterology and Hepatology Research Center, Institute of Basic and Clinical Physiology Sciences, Kerman University of Medical Sciences, Kerman, Iran

**Keywords:** Hepatic cirrhosis, Chronic lymphocytic leukemia, Portal hypertension, Comorbidity, Case report

## Abstract

**Background:**

Chronic lymphocytic leukemia, a common B-cell malignancy in the elderly, is characterized by lymphocytosis, lymphadenopathy, and cytopenia. Concurrently, hepatic cirrhosis, defined by liver fibrosis and portal hypertension, often leads to overlapping clinical features such as splenomegaly and cytopenia, complicating the diagnosis and management of chronic lymphocytic leukemia.

**Case presentation:**

We present an 84-year-old Iranian male patient with established hepatic cirrhosis who presented with abdominal pain, massive splenomegaly, lymphocytosis, anemia, and cachexia. Initial evaluations revealed lymphocyte-dominant leukocytosis and smudge cells. Immunophenotyping confirmed chronic lymphocytic leukemia (cluster of differentiation 5+, cluster of differentiation 19+, cluster of differentiation 20+, cluster of differentiation 23+). Imaging demonstrated liver cirrhosis and portal hypertension with esophageal varices.

**Conclusion:**

This case highlights the diagnostic complexities of concurrent chronic lymphocytic leukemia and hepatic cirrhosis, necessitating a multidisciplinary approach and targeted diagnostic strategies to differentiate overlapping clinical manifestations. It underscores the importance of individualized treatment in such complex patients to optimize outcomes and enhance quality of life.

## Introduction

Chronic lymphocytic leukemia (CLL) is a clonal malignancy of B lymphocytes and represents the most common type of leukemia in adults, particularly affecting the elderly population. CLL is characterized by an accumulation of small, mature-appearing lymphocytes in the blood, bone marrow, and lymphoid tissues, which can lead to variable clinical presentations ranging from asymptomatic lymphocytosis to symptomatic disease with lymphadenopathy, splenomegaly, and cytopenia [1, 2]. While CLL typically follows an indolent course, its management becomes complex in the presence of significant comorbidities, such as hepatic cirrhosis, which can significantly complicate its diagnosis by masking or exacerbating typical clinical features [3].

Hepatic cirrhosis, characterized by liver fibrosis and architectural distortion, results from diverse chronic liver injuries (for example, viral hepatitis, alcohol use, metabolic liver diseases) [4]. Cirrhosis is frequently complicated by portal hypertension, which can lead to splenomegaly and hypersplenism, resulting in anemia and thrombocytopenia [5]. These hematologic abnormalities often overlap with those observed in CLL, further complicating differential diagnosis. Furthermore, the systemic symptoms of cirrhosis, such as fatigue and weight loss, may be compounded by the metabolic derangements associated with liver dysfunction, further obscuring the clinical picture [6].

Diagnosing CLL concurrently with hepatic cirrhosis presents unique diagnostic challenges, necessitating a comprehensive, multidisciplinary approach due to overlapping clinical presentations. Advanced diagnostic tools, such as flow cytometry, are essential for distinguishing CLL from other lymphoproliferative disorders and for ruling out conditions such as non-Hodgkin lymphoma (NHL) [7, 8]. Immunophenotyping plays a critical role in identifying the characteristic markers of CLL, such as cluster of differentiation (CD)5, CD19, CD20, and CD23, which aid in confirming the diagnosis and guiding treatment decisions [9]. Furthermore, iron profile evaluation is crucial to exclude hemochromatosis as a potential etiology of cirrhosis, ensuring targeted treatment of the underlying pathology [10].

This case report presents an 84-year-old patient with concurrent hepatic cirrhosis and CLL, highlighting the intricate clinical presentation and the diagnostic challenges encountered. The patient exhibited significant abdominal pain, massive splenomegaly, lymphocyte-dominant leukocytosis, anemia, thrombocytopenia, and systemic symptoms such as anorexia and cachexia. These findings necessitated a systematic evaluation to delineate the underlying etiology and guide appropriate management [11]. Through a detailed analysis of clinical, laboratory, and imaging findings, this report underscores the importance of a tailored diagnostic approach in managing patients with complex comorbidities [12].

The interplay between CLL and hepatic cirrhosis in this patient emphasizes the necessity of a multidisciplinary approach. Collaboration among hematology, gastroenterology, and primary care is essential to address the multifaceted needs of patients with such overlapping conditions. This case report aims to contribute to the understanding of managing CLL in patients with significant liver disease, providing insights into diagnostic strategies and therapeutic interventions that can optimize patient outcomes [13].

## Case report

An 84-year-old male of Iranian ethnicity presented to our gastroenterology clinic with a 1-month history of persistent abdominal pain and fullness. The pain was characterized as constant, non-colicky, and non-positional, localized to the epigastric region and right upper quadrant, with radiation to the right shoulder, and was not associated with food intake or bowel movements.

Significant anorexia, nausea, and intermittent vomiting were also reported. Initially, vomiting occurred three times per week, containing undigested food particles. After 2 weeks, the emesis evolved to clear fluid with streaks of blood, prompting concern for gastrointestinal bleeding.

The patient reported no history of diarrhea or melena, and stool consistency was normal without constipation. His medical history was significant for hepatic cirrhosis of unknown etiology, diagnosed 2 years prior, which was managed with lactulose for hepatic encephalopathy prophylaxis, propranolol for portal hypertension, and spironolactone for ascites. These interventions aimed to control symptoms and complications of his underlying liver disease. In addition, 4 years before presentation, he underwent cholecystectomy for symptomatic choledocholithiasis, resolving the acute gallbladder issues. He also had a history of chronic obstructive pulmonary disease (COPD), which was managed with corticosteroid inhalers, providing symptomatic relief for his respiratory condition.

Social history included a 30 pack-year smoking history and daily oral opium use. He denied alcohol abuse or the use of other illicit drugs. No known allergies to medications, foods, or environmental factors were reported. Family history was unremarkable for similar conditions.

Examination revealed an ill-appearing patient with cachexia with marked pallor and temporal muscle wasting. Vital signs showed hypotension (90/60 mmHg), a pulse of 72 beats per minute, tachypnea (25 breaths per minute), and fever (38.9 °C). Oxygen saturation was 90% on room air.

Head and neck examination showed pale conjunctivae and anicteric sclerae. Chest auscultation revealed bilateral lower lobe crackles. Cardiac examination was unremarkable. Abdominal examination demonstrated asymmetry with left-sided prominence. Palpation elicited tenderness in the epigastric and right upper quadrants without guarding. The liver was palpably nodular, consistent with cirrhosis, and the spleen was massively enlarged with an impalpable inferior border. Motor strength was 4/5, indicating mild generalized weakness.

This comprehensive physical examination highlights the complexity of the patient’s clinical presentation, with findings suggestive of anemia, possible pulmonary involvement, and significant hepatic and splenic abnormalities. These findings, in conjunction with the patient’s history and presenting symptoms, necessitate further diagnostic evaluation to elucidate the underlying etiology and guide appropriate management.

Initial laboratory investigations, summarized in Table 1, included a complete blood count (CBC) with differential, liver, and renal function tests; coagulation profile; electrolytes; fasting blood sugar (FBS); creatine phosphokinase (CPK); lactate dehydrogenase (LDH); serum ammonia; and alpha-fetoprotein (AFP).

**Table 1:** Laboratory results on admission

Laboratory test	Result	Normal range (units)
White blood cell count (WBC)	31.8	4.0–11.0 (× 10^3^/L)
Hemoglobin (Hb)	11	13.8–17.2 (g/dL) (male)
Mean corpuscular volume (MCV)	83	80–100 (fL)
Mean corpuscular hemoglobin (MCH)	24.2	27–33 (pg)
Mean corpuscular hemoglobin concentration (MCHC)	29.2	32–36 (g/dL)
Platelet count (PLT)	95	150–450 (× 10^3^/L)
Lymphocyte	70.6	20–40 (%)
Neutrophile	23.5	40–60 (%)
Mixed	5.9	0–10 (%)
Aspartate aminotransferase (AST)	55	10–40 (U/L)
Alanine aminotransferase (ALT)	23	7–56 (U/L)
Alkaline phosphatase (ALKPH)	729	44–147 (U/L)
Total bilirubin (BILI T)	1.2	0.1–1.2 (mg/dL)
Direct bilirubin (BILI D)	0.5	0.0–0.3 (mg/dL)
Prothrombin time (PT)	16.3	11–13.5 (seconds)
Partial thromboplastin time (PTT)	29	25–35 (seconds)
International normalized ratio (INR)	1.27	0.8–1.2
Sodium (Na)	141	135–145 (mmol/L)
Potassium (K)	4.2	3.5–5.0 (mmol/L)
Urea	29	7–20 (mg/dL)
Creatinine	0.9	0.6–1.2 (mg/dL)
Fasting blood sugar (FBS)	91	70–100 (mg/dL)
Lactate dehydrogenase (LDH)	377	140–280 (U/L)
Creatine phosphokinase (CPK)	107	20–200 (U/L)
Serum ammonia levels	18	15–45 (µmol/L)
Alpha-fetoprotein (AFP)	3	< 10 (ng/ml)
PH	7.37	7.35–7.45
Partial pressure of carbon dioxide (PCO2)	52.8	35–45 (mmHg)
Bicarbonate (HCO3)	30.2	22–26 (mmol/L)

Abdominal ultrasonography, prompted by significant splenomegaly, revealed a normal-sized liver with nodular appearance and irregular borders, consistent with cirrhosis and no focal hepatic mass. Cholecystectomy status was noted. The common bile duct (CBD) was dilated to 13 mm, raising suspicion for biliary obstruction. Mild free fluid in the left subphrenic space and engorged mesenteric vessels suggested portal hypertension. Both kidneys were normal in size and consistency. The bladder was filled with urine and appeared normal, while the prostate was enlarged, consistent with benign prostatic hyperplasia.

Despite CBD dilation on ultrasound, bilirubin levels were normal. Magnetic resonance cholangiopancreatography (MRCP) was thus performed to evaluate for biliary and pancreatic obstruction or masses, which revealed no evidence of intra- or extrahepatic biliary ductal obstruction, strictures, or mass, effectively excluding cholangiocarcinoma or other neoplastic processes.

Given the history of bloody vomitus, an upper gastrointestinal endoscopy was performed. It revealed nonbleeding grade 2 esophageal varices (5–10 mm diameter, occupying < 50% circumference of distal esophagus) without red color signs. Notably, there were no red color signs observed in the varices located in the middle and lower thirds of the esophagus. The Z-line was visualized without abnormalities. Examination of the cricopharynx and the upper third of the esophagus revealed no pathological changes. Endoscopic band ligation was performed on the varices to prevent future hemorrhage, completed without complications.

Additionally, a single 10 mm polyp was identified in the fundus of the stomach. Multiple biopsies were obtained from this gastric polyp to facilitate histopathological evaluation and determine the nature of the lesion. The pathology report of the biopsy revealed that the gastric polyp was a benign hyperplastic polyp, with no evidence of dysplasia or malignancy.

On the basis of the comprehensive clinical presentation, laboratory examinations, and imaging findings of this 84-year-old patient, a spectrum of differential diagnoses arose from the complex interplay of his symptoms and medical history. The patient presented with significant abdominal pain, massive splenomegaly, lymphocyte-dominant leukocytosis, anemia, and thrombocytopenia, alongside systemic symptoms such as anorexia, fever, and cachexia. These findings, coupled with a background of hepatic cirrhosis and a history of gastrointestinal bleeding, necessitated a thorough differential diagnosis process to elucidate the underlying etiology. Below, we explore the potential differential diagnoses, considering the patient’s intricate clinical scenario:

1. Chronic lymphocytic leukemia (CLL): The predominant lymphocytosis, presence of smudge cells, and systemic symptoms strongly suggest CLL. This hematological malignancy is characterized by the accumulation of small, mature lymphocytes, leading to cytopenia and organomegaly, particularly splenomegaly. The patient’s cachexia and fever are consistent with the advanced stages of CLL, which often presents with such constitutional symptoms due to the high leukemic burden and immune dysfunction [14].
2. Myelodysplastic syndromes (MDS): MDS should be considered due to the patient’s age and hematological abnormalities, including anemia and thrombocytopenia. MDS is characterized by ineffective hematopoiesis and can progress to acute leukemia. Although lymphocyte predominance is atypical, the cytopenia warrants investigation for this disorder [15].
3. Non-Hodgkin lymphoma (NHL): Consideration of NHL is warranted given the lymphocyte-dominant leukocytosis and splenomegaly. NHL can present with systemic symptoms such as fever, weight loss, and night sweats, alongside lymphadenopathy and organomegaly. The differentiation from CLL often relies on histological and immunophenotypic analyses [8].
4. Hemochromatosis: This genetic condition leads to excessive iron deposition, potentially causing cirrhosis. It can present with diabetes, skin changes, and arthralgia. While the patient’s presentation primarily suggested a hematological malignancy, iron studies were essential to rule out hemochromatosis as a contributing factor [11].
5. Hepatocellular carcinoma (HCC): With a background of hepatic cirrhosis, the patient is at increased risk for HCC. The nodular liver appearance on imaging could indicate neoplastic changes. HCC typically presents with abdominal pain, weight loss, and cachexia, symptoms observed in this patient. The surveillance of patients with cirrhosis for HCC is critical due to its potential for rapid progression and impact on prognosis [16].
6. Portal hypertension with esophageal varices: The endoscopic finding of esophageal varices is indicative of portal hypertension, likely secondary to cirrhosis. This condition could explain the splenomegaly and contribute to the observed anemia and thrombocytopenia through splenomegaly. The gastrointestinal bleeding history aligns with variceal hemorrhage, a common complication of portal hypertension [5].
7. Autoimmune hepatitis: Although the patient lacks jaundice, the cirrhosis of unknown etiology and systemic symptoms could suggest autoimmune hepatitis. This condition can cause chronic liver inflammation, leading to cirrhosis, and may present with fatigue, anorexia, and elevated liver enzymes. Serological testing and liver biopsy are essential for diagnosis [17].

**Fig. 1:**
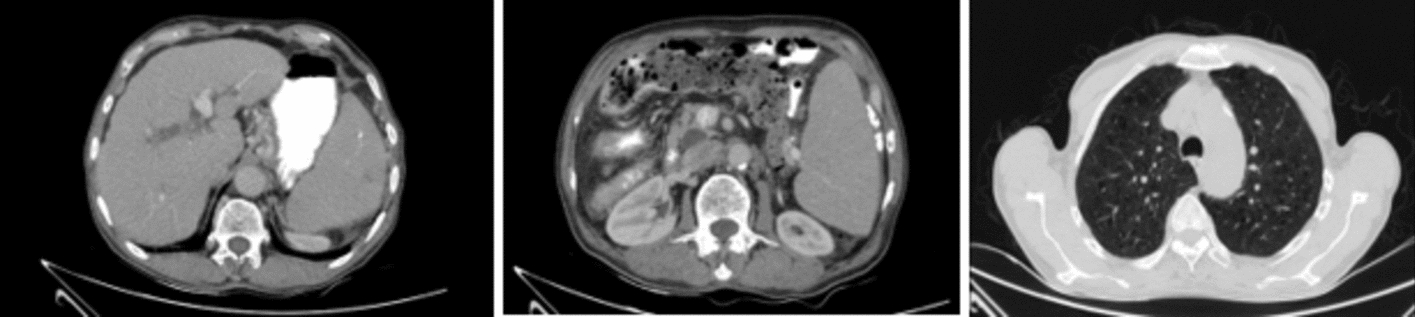
**A** Peripheral blood findings: smear prepared at ×1000 magnification under an oil immersion lens and stained with Giemsa. Red blood cell morphology reveals anisocytosis, mild poikilocytosis, mild ovalocytosis, and the presence of a few target cells. White blood cell morphology shows a markedly elevated count with the following differential: polymorphonuclear cells (14%), lymphocytes (47%), eosinophils (2%), bands (2%), metamyelocytes (2%), atypical lymphocytes (31%), and prolymphocytes (2%). Smudge cells are observed, along with large platelets. **B** Bone marrow aspiration: specimen examined at ×1000 magnification using Giemsa staining. The aspirate differential reveals myeloid cells (26%), erythroid cells (7%), lymphoid cells (64%), and blasts (3%). Smudge cells are noted, along with significant infiltration by small lymphoid cells. Flow cytometry analysis confirms that the lymphoid cells express cluster of differentiation 5, cluster of differentiation 19, and cluster of differentiation 20, while being negative for FMC7 and cluster of differentiation 22. Megakaryocytes are also observed. **C** Bone marrow biopsy: histological sections prepared at ×400 magnification and stained with hematoxylin and eosin. The biopsy demonstrates 80% cellularity with interstitial and focal para- and non-paratrabecular infiltration by small lymphoid cells. Megakaryocytes are present

Clinical assessment and laboratory investigations, including massive splenomegaly, lymphocyte-dominant leukocytosis, anemia, thrombocytopenia, and systemic symptoms (anorexia, fever, cachexia), strongly suggested a hematological malignancy. Peripheral blood smear analysis revealed a predominance of mature lymphocytes and smudge cells, characteristic findings of CLL (Fig. 1), an elderly onset leukemia marked by functionally incompetent lymphocyte accumulation.

Bone marrow aspiration was performed to confirm the diagnosis and assess the extent of malignant lymphocyte infiltration and marrow-suppression-induced cytopenia, as well as to exclude other hematologic malignancies. Cytological examination and flow cytometry of the aspirate identified characteristic CLL immunophenotypic markers. Bone marrow analysis revealed diffuse infiltration by small, mature lymphocytes consistent with CLL, corroborating peripheral blood findings (Fig. 1).

The constellation of clinical and laboratory findings, including the significant splenomegaly, lymphocyte-predominant leukocytosis, and cytopenia, aligns with the typical presentation of CLL. The patient’s systemic symptoms, such as anorexia and cachexia, further support this diagnosis, as they are often associated with advanced disease stages or high tumor burden. The presence of smudge cells on the peripheral blood smear is a classic laboratory finding in CLL, resulting from the fragility of the leukemic lymphocytes during slide preparation.

To exclude other differential diagnoses, an iron profile (serum ferritin, transferrin saturation, total iron binding capacity) was performed to assess for hemochromatosis. These tests were unremarkable, ruling out iron accumulation as a contributor to liver pathology and systemic symptoms.

Furthermore, the immunophenotyping studies were crucial in differentiating between CLL and other lymphoproliferative disorders such as NHL. Flow cytometry analysis of the peripheral blood demonstrated a clonal population of B-cells with the characteristic immunophenotype of CLL, including coexpression of CD5–CD19 and CD20. The absence of markers typically associated with NHL further corroborated the diagnosis of CLL. These targeted diagnostic approaches allowed for the exclusion of other potential hematological and metabolic disorders, leading to a definitive diagnosis of CLL and guiding the subsequent management plan for this patient.

In this case, the diagnosis of CLL was established on the basis of the integration of clinical presentation, laboratory findings, and peripheral blood smear morphology. The patient’s significant splenomegaly and cytopenia necessitate further evaluation to assess the extent of disease involvement and guide therapeutic decision-making.

## Discussion

Our patient’s presentation illustrates the complexities of CLL, particularly when coexisting with hepatic cirrhosis. CLL is a clonal malignancy of B lymphocytes, characterized by the accumulation of mature lymphocytes in the blood, bone marrow, and lymphoid tissues. It is the most common form of leukemia in adults, particularly affecting the elderly population [18, 19]. The disease often presents with lymphocytosis, lymphadenopathy, and splenomegaly, and can be complicated by cytopenia due to bone marrow infiltration. The management of CLL becomes particularly challenging when it coexists with other significant medical conditions, such as hepatic cirrhosis, which can mask or exacerbate the clinical features of CLL [4].

The management of CLL in patients with concurrent hepatic cirrhosis presents significant challenges due to overlapping clinical features and the complexity of treatment decisions. This case of an 84-year-old male patient with CLL and hepatic cirrhosis underscores these challenges, emphasizing the need for a nuanced approach that considers both the hematological and hepatic components of the patient’s condition.

Staging in chronic lymphocytic leukemia (CLL) is essential for assessing disease prognosis and guiding treatment decisions. Two widely used staging systems for CLL are the Rai and Binet systems. Both systems provide valuable prognostic information, with higher stages being associated with reduced survival and increased risk of disease progression [1, 2].

**Fig. 2:**
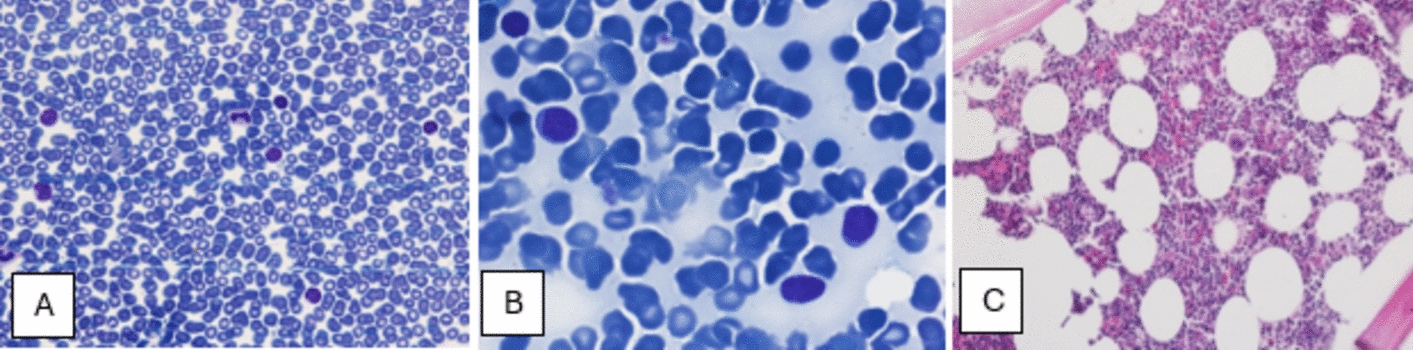
Abdominal computed tomography findings: abdominal computed tomography scan revealed massive splenomegaly and the absence of the gallbladder, consistent with a prior cholecystectomy. The common bile duct was dilated to 13 mm. Scattered calcifications were noted within the pancreas. Additionally, paraaortic lymph nodes were identified, with a maximum short-axis diameter of 7 mm. Chest computed tomography findings: revealed bilateral centriacinar emphysema, characterized by focal destruction of the central portions of secondary pulmonary lobules. Notably, there were no radiological signs of lymphadenopathy in the thoracic region

When staging CLL, imaging plays a critical role. In this patient, computed tomography (CT) scan of the abdomen and chest was performed to assess the extent of disease. The chest CT scan revealed bilateral centriacinar emphysema, characterized by focal destruction of the central portions of secondary pulmonary lobules. Scattered areas of atelectasis were observed, along with vascular calcifications involving the coronary vessels, indicative of underlying atherosclerotic changes. Notably, there were no radiological signs of lymphadenopathy in the thoracic region. Abdominal CT revealed paraaortic lymph nodes with a maximum short axis diameter of 7 mm (Fig. 2).

This finding, coupled with splenomegaly, aligns with Rai stage II CLL, indicating an intermediate risk level [1, 2]. This stage is characterized by lymphocytosis and organomegaly but without anemia or thrombocytopenia severe enough to classify as a higher stage.

Targeted therapies, such as ibrutinib, a Bruton’s tyrosine kinase (BTK) inhibitor, are preferred due to their efficacy and more manageable safety profile in elderly patients. Ibrutinib exerts its therapeutic effect by irreversibly binding to BTK, thereby inhibiting B-cell receptor signaling and subsequent proliferation and survival of malignant B cells [12]. Alternatively, venetoclax, a BCL-2 inhibitor, is considered for those intolerant to ibrutinib or with contraindications to its use, as it effectively induces apoptosis in CLL cells [9]. These agents are less myelosuppressive than traditional chemotherapy, which is crucial for patients with cirrhosis-related hypersplenism that contributes to cytopenia [20].

Given these considerations, treatment with ibrutinib was initiated in this patient, administered orally at a standard dose of 420 mg daily. The treatment was well tolerated, with no significant adverse effects reported.

Following the initiation of oral ibrutinib at a standard dose of 420 mg daily, the patient was closely monitored through regular follow-up visits conducted every 4 weeks. During these visits, a comprehensive assessment included complete blood counts, liver function tests, and evaluation of clinical symptoms to ascertain treatment efficacy and safety. Over the subsequent 6 months, imaging studies, specifically CT scans of the abdomen, were repeated every 6 months to monitor for disease progression, assess lymph node size, and evaluate splenomegaly. Throughout this period, the patient reported good tolerability to ibrutinib with no significant adverse effects. Clinically, there was a gradual improvement in his systemic symptoms, including a reduction in abdominal pain and a decrease in splenomegaly, accompanied by stabilization of his hematological parameters. Liver function tests remained stable, and no new complications related to either CLL or cirrhosis were observed during the follow-up period.

This management strategy aligns with current guidelines and literature, emphasizing the importance of individualized treatment plans in CLL, particularly in patients with comorbid conditions. Byrd *et al*. demonstrated the effectiveness of ibrutinib in treating elderly patients with CLL, supporting its use in this demographic [12].

## Conclusion

This case report underscores the complexity of managing CLL in patients with concurrent hepatic cirrhosis. The coexistence of these conditions complicates both diagnosis and treatment due to overlapping symptoms and the need for careful therapeutic choices. In this patient, ibrutinib was effectively used, demonstrating its suitability as a targeted therapy with a favorable safety profile for elderly patients with significant comorbidities. Regular follow-up ensured the monitoring of treatment efficacy and the management of potential side effects. This case highlights the necessity of individualized care and a multidisciplinary approach, emphasizing the importance of integrating novel therapies to optimize outcomes and improve quality of life in similar complex clinical scenarios.

## Limitations

As a single case report, this study’s findings may not be generalizable to all patients with concurrent CLL and hepatic cirrhosis. The retrospective nature also inherently limits the ability to control for all variables.

## Key clinical message

This case report highlights the diagnostic complexities of concurrent chronic lymphocytic leukemia (CLL) and hepatic cirrhosis. Overlapping clinical features necessitate a multidisciplinary approach and advanced diagnostics for accurate differentiation. Individualized treatment strategies are crucial to optimize outcomes and enhance the quality of life in such complex patients.

## Data Availability

The datasets used or analyzed during the current study are available from the corresponding author upon reasonable request. This includes anonymized patient data to ensure confidentiality while allowing for scientific scrutiny and verification of results.

## Abbreviations

CLL: Chronic lymphocytic leukemia
NHL: Non-Hodgkin lymphoma
COPD: Chronic obstructive pulmonary disease
CBC: Complete blood count
FBS: Fasting blood sugar
CPK: Creatine phosphokinase
LDH: Lactate dehydrogenase
AFP: Alpha-fetoprotein
CBD: Common bile duct
MRCP: Magnetic resonance cholangiopancreatography
MDS: Myelodysplastic syndromes
HCC: Hepatocellular carcinoma
CT: Computed tomography
